# Navigating the challenges of catheter ablation in patients with comorbid alcohol use disorder

**DOI:** 10.1186/s12872-025-05356-6

**Published:** 2025-12-12

**Authors:** Yue Liu, Jun Ye, Bing Zeng, Jianming Song, Yaoyang Huo, Fang Yu, Lixin Chen

**Affiliations:** 1 Department of Ultrasound Medicine, First Affiliated Hospital of Gannan Medical University, Ganzhou, Jiangxi Province 341000 China; 2 Joint Surgery Department, People’s Hospital of Ganzhou, Ganzhou, Jiangxi Province 341000 China; 3 The Second School of Clinical Medicine, Southern Medical University, Guangzhou, Guangdong Province 510515 China

**Keywords:** Alcohol use disorder, Cardiac arrhythmias, Catheter ablation, Postoperative complications, Mortality paradox

## Abstract

**Background:**

Alcohol use disorder (AUD) is a significant risk factor for cardiac arrhythmias, contributing to arrhythmogenesis through direct myocardial toxicity and electrical remodeling. The present study evaluated the impact of AUD on clinical and economic outcomes following catheter ablation.

**Methods:**

The National Inpatient Sample (2010–2019) was used to identify and analyze adults who underwent catheter ablation. Patients were stratified based on the presence of an AUD diagnosis and balanced using a 1:5 propensity score matching for demographics and comorbidities. Outcomes were assessed using logistic regression, Pearson chi-square test, and the Wilcoxon rank-sum test.

**Results:**

Among the 109,226 patients evaluated, 3,113 (2.85%) had AUD. The AUD cohort was younger, predominantly male, and had higher rates of smoking (40.6% vs. 16.5%), drug abuse (10.3% vs. 5.5%), and liver disease (11.3% vs. 7.8%). After matching, AUD was associated with a longer median length of stay (4.0 vs. 3.0 days, *P* < 0.001), higher costs ($118,427 vs. $116,503, *P* < 0.001), and a significantly elevated risk of gastrointestinal complications, particularly esophageal ulcers (0.4% vs. 0.1%, *P* = 0.006). Interestingly, in-hospital mortality was lower in the AUD group (0.6% vs. 1.4%, *P* < 0.001).

**Conclusions:**

The distinct clinical profile and higher risk of specific post-ablation complications among patients with AUD reflect the need for specialized preoperative optimization. Furthermore, the paradoxically lower in-hospital mortality observed in this cohort warrants further investigation and may reflect selection bias favoring a more resilient AUD subgroup.

**Supplementary Information:**

The online version contains supplementary material available at 10.1186/s12872-025-05356-6.

## Introduction

The association between alcohol consumption and cardiac arrhythmias has been recognized for more than a century, with reports of alcohol’s cardiotoxic effects dating back to the 1800 s [1]. This relationship typically exhibits a J-shaped dose-response pattern, in which heavy drinking substantially increases the likelihood of developing hypertension, alcoholic cardiomyopathy, and various arrhythmias, particularly atrial fibrillation (AF) [2]. Moreover, individuals with alcohol-related cardiac disease exhibit an increased susceptibility to life-threatening ventricular arrhythmias, such as ventricular tachycardia and sudden cardiac death, with these risks correlating directly with lifetime alcohol exposure [3].

Since the 1990 s, catheter ablation has remained the cornerstone therapy for cardiac rhythm disorders. Initially applied to simple supraventricular tachycardias, this technique is now a primary treatment approach for complex arrhythmias, including atrial fibrillation (AF) and ventricular tachycardia (VT) [4]. Nevertheless, while comprehensive data on ablation outcomes exist, real-world evidence remains limited for certain high-risk patient subgroups. Specifically, patients with alcohol use disorder (AUD) represent a challenging and understudied population who face heightened periprocedural risks. These risks are due to alcohol’s direct cardiotoxic effects and substantial comorbidity burden [5]. Notably, a significant gap remains in understanding periprocedural risks, including complication rates, length of stay, costs, and in-hospital mortality, specifically in arrhythmia patients with coexisting AUD.

We surmised that patients with AUD have a distinct risk profile and experience higher perioperative complication rates following catheter ablation compared with patients without AUD. To that end, we conducted a retrospective cohort study using a comprehensive, nationwide database. Our study aimed to characterize the impact of AUD on in-hospital outcomes post-ablation, with the goal of refining preoperative risk stratification and guiding personalized management for this population.

## Methods

### Data source

Data from the Nationwide Inpatient Sample (NIS) for 2010–2019 were analyzed [6]. As a cornerstone of the Healthcare Cost and Utilization Project (HCUP) sponsored by the Agency for Healthcare Research and Quality (AHRQ), the NIS represents the largest publicly available all-payer inpatient database in the United States (U.S.). It constitutes a 20% stratified sample of U.S. community hospitals, encompassing approximately 8 million hospitalizations annually from over 1,000 facilities across 44 states. Of note, this sampling frame captures around 90% of the nation’s nonprofit teaching hospitals. As the NIS contains publicly available, de-identified data, this study was exempt from institutional review board approval [7].

### Study cohort

The study utilized the NIS database from 2010 to 2019, which contained data on 72,950,400 hospitalizations. From this initial pool, we identified patients with a primary diagnosis of cardiac arrhythmia who underwent catheter ablation using the relevant International Classification of Diseases (ICD-9-CM and ICD-10-CM) procedure codes (Table A).Herein, we included patients with atrial fibrillation, atrial flutter, supraventricular tachycardia, ventricular tachycardia, Wolff-Parkinson-White syndrome, premature contractions, or unspecified arrhythmias (Table 1). To avoid confounding the attribution of periprocedural complications, we excluded patients who underwent concurrent pacemaker or implantable cardioverter-defibrillator (ICD) implantation.

**Table 1:** Proportions of specific arrhythmia subtypes ablated in the AUD study population

Arrhythmia Subtype	After matching		
	non-AUD	AUD	*p* value
Catheter ablation procedure	13,171(96.6%)	2878(95.4%)	0.001
Atrial Fibrillation	8425(61.8%)	1786(59.2%)	0.008
Atrial flutter	6232(45.7%)	1626(53.9%)	<0.001
Supraventricular tachycardia	3691(27.1%)	844(28.0%)	0.312
Ventricular tachycardia	2761(20.3%)	481(15.9%)	<0.001
Wolff-Parkinson-White syndrome/preexcitation syndrome	492(3.6%)	112(3.7%)	0.782
other premature beats	267(2.0%)	62(2.1%)	0.729

*AUD* Alcohol Use Disorder

**Fig. 1:**
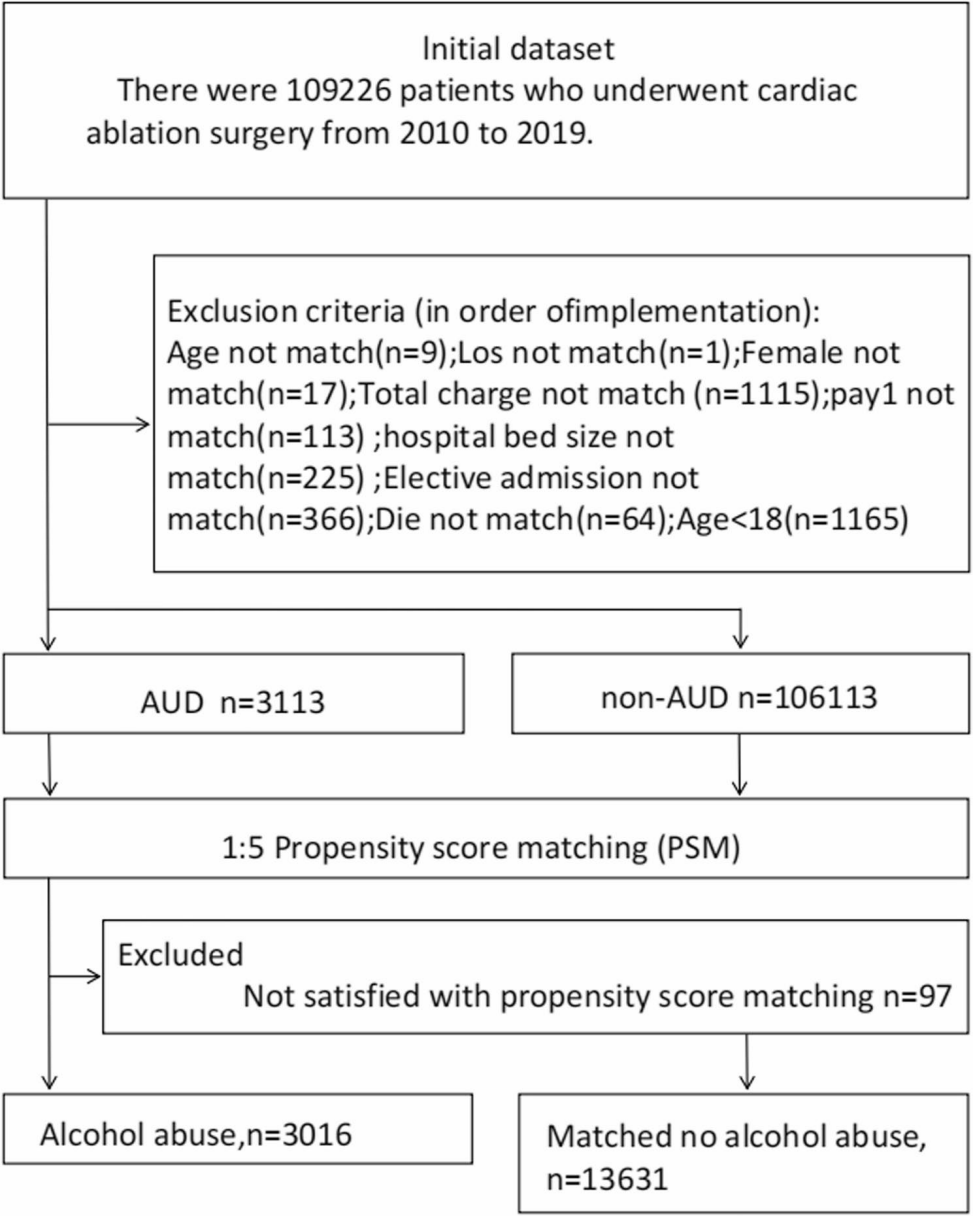
Patient Selection Flowchart

From an initial cohort of 109,226 patients who underwent ablation, we excluded 1,165 individuals for being under 18 years of age or having non-elective hospitalizations. Next, 1:5 propensity score matching (PSM) (caliper width = 0.01) was performed based on baseline characteristics, achieving an exclusion rate of 3.1%. Consequently, a final analytical cohort of 16,647 patients was achieved and they were further categorized into two groups: those with a documented history of AUD (*n* = 3,016) and those without such a history (*n* = 13,631) (Fig. 1). A recognized limitation of using administrative data in this context is that the International Classification of Diseases (ICD) codes for alcohol use disorder (AUD) do not differentiate between past and current use.

The NIS database provides comprehensive patient-level data on demographics (age, sex, race), insurance status, hospital characteristics, and pertinent comorbidities. Here, our primary outcome was in-hospital and all-cause mortality. Secondary outcomes included length of stay (LOS), total healthcare costs, and perioperative complications, which were systematically identified using standardized ICD diagnostic codes (Table B). All measured baseline covariates were included in the model to balance the cohorts (Table 2).

**Table 2:** Demographic and comorbidities characteristic of study cohorts

Demographic/comorbidity	Before matching			After matching		
	non-AUD	AUD	*p* value	non-AUD	AUD	*p* value
Total (n = count)	106,113	3113		13,631	3016	
Total incidence (%)	2.85(3113/109226)			18.12(3,016/16,647)		
Age (median, years)	67.0 (58.0, 75.0)	60.0(53.0, 67.0)	0.000	62.0 (52.0,71.0)	60.0(53.0, 68.0)	< 0.001
Age group (%)						
18–44	28.8	47.2	< 0.001	43.6	46.6	< 0.001
45–64	29.1	33.7		28.5	33.9	
65–74	27.7	16.0		19.7	16.4	
≥ 75	14.5	3.1		8.1	3.2	
Gender (%)						
Male	60.6	86.9	< 0.001	86.8	86.6	0.748
Female	39.4	13.1		13.2	13.4	
Race (%)						
White	74.0	67.0	< 0.001	68.2	67.6	0.505
Black	9.4	16.3		14.6	15.9	
Hispanic	5.3	6.6		6.5	6.6	
Asian or Pacific Islander	1.5	0.9		0.9	0.9	
Native American	0.3	0.7		0.6	0.7	
Other	9.5	8.5		8.2	8.6	
LOS (median, d)	3.0 (2.0–6.0)	4.0 (3.0–8.0)	< 0.001	4.0 (2.0–7.0)	4.0 (2.0–8.0)	< 0.001
TOTCHG (median, $)	101994.0 (66902.0–155382.5.0.5)	110971.0 (72216.0–172424.0.0.0)	< 0.001	116503.0 (77968.7–181360.0.7.0)	118428.1 (77900.7–183408.7.7.7)	< 0.001
Type of insure (%)						
Medicare	58.9	40.4	< 0.001	42.7	41.2	0.001
Medicaid	6.1	17.5		14.3	16.7	
Private insurance	30.4	29.2		31.7	29.8	
Self-pay	1.9	7.4		6.0	6.9	
No charge	0.2	1.3		1.0	1.3	
Other	2.4	4.1		4.2	4.0	
Bed size of hospital (%)						
Small	8.4	8.9	0.363	8.7	8.9	0.862
Medium	21.1	21.8		21.4	21.8	
Large	70.5	69.3		69.9	69.4	
Elective admission (%)	40.8	19.8	< 0.001	21.3	20.4	0.286
Type of hospital (teaching %)	78.9	81.9	< 0.001	81.1	81.9	0.275
Location of hospital (urban, %)	97.4	97.9	0.125	97.8	97.8	0.838
Region of hospital (%)						
Northeast	23.8	21.8	0.048	22.4	22.1	0.827
Midwest or North Central	21.5	21.8		21.9	21.6	
South	39.6	40.2		39.3	40.2	
West	15.2	16.2		16.4	16.1	
Preoperative comorbidities						
Acquired immune deficiency syndrome	215(0.2%)	12 (0.4%)	0.027	42(0.3%)	12 (0.4%)	0.433
Deficiency anemia	8057 (7.6%)	299 (9.6%)	<0.001	1246 (9.1%)	284 (9.4%)	0.636
Rheumatoid arthritis/collagen vascular diseases	2542 (2.4%)	52 (1.7%)	0.009	257 (1.9%)	51(1.7%)	0.473
Chronic blood loss anemia	485 (0.5%)	13 (0.4%)	0.747	51 (0.4%)	13 (0.4%)	0.648
Congestive heart failure	20,614 (19.4%)	832 (26.7%)	<0.001	3451(25.3%)	798 (26.5%)	0.193
Chronic pulmonary disease	23,359 (22.0%)	991 (31.8%)	<0.001	4186(30.7%)	946 (31.4%)	0.480
Coagulopathy	5011 (4.7%)	359(11.5%)	<0.001	1296(9.5%)	322(10.7%)	0.050
Depression	8293 (7.8%)	362 (11.6%)	<0.001	1404 (10.3%)	340 (11.3%)	0.114
Diabetes, uncomplicated	21,108 (19.9%)	448 (14.4%)	<0.001	2043(15.0%)	437 (14.5%)	0.487
Diabetes with chronic complications	8125(7.7%)	208 (6.7%)	0.043	934(6.9%)	199 (6.6%)	0.616
Drug abuse	1283 (1.2%)	370(11.9%)	<0.001	753(5.5%)	311(10.3%)	<0.001
Hypertension	71,763 (67.6%)	2192 (70.4%)	0.001	9590 (70.4%)	2121 (70.3%)	0.974
Hypothyroidism	15,510 (14.6%)	225 (7.2%)	<0.001	1011 (7.4%)	219 (7.3%)	0.767
Liver disease	2314 (2.2%)	413(13.3%)	<0.001	1069 (7.8%)	342(11.3%)	<0.001
Lymphoma	702(0.7%)	10 (0.3%)	0.020	52(0.4%)	10(0.3%)	0.684
Fluid and electrolyte disorders	17,841 (16.8%)	874 (28.1%)	<0.001	3491 (25.6%)	816 (27.1%)	0.101
Metastatic cancer	581 (0.5%)	13(0.4%)	0.331	67(0.5%)	13(0.4%)	0.664
Other neurological disorders	3544 (3.3%)	155 (5.0%)	<0.001	656 (4.8%)	144(4.8%)	0.930
Obesity	20,291 (19.1%)	636 (20.4%)	0.068	2866(21.0%)	624 (20.7%)	0.682
Paralysis	847 (0.8%)	26 (0.8%)	0.819	110 (0.8%)	24 (0.8%)	0.950
Peripheral vascular disorders	7913(7.5%)	250 (8.0%)	0.230	1058(7.8%)	241 (8.0%)	0.671
Psychoses	1865(1.8%)	151 (4.9%)	<0.001	533(3.9%)	138 (4.6%)	0.093
Pulmonary circulation disorders	4087 (3.9%)	143 (4.6%)	0.034	604(4.4%)	135 (4.5%)	0.913
Renal failure	18,109 (17.1%)	420 (13.5%)	<0.001	1898 (13.9%)	406 (13.5%)	0.506
Solid tumor without metastasis	1517 (1.4%)	46 (1.5%)	0.824	179(1.3%)	45(1.5%)	0.440
Peptic ulcer disease excluding bleeding	188 (0.2%)	9 (0.3%)	0.147	40 (0.3%)	8(0.3%)	0.794
Valvular disease	9557 (9.0%)	307 (9.9%)	0.101	1288 (9.4%)	291 (9.6%)	0.735
Weight loss	2307(2.2%)	125(4.0%)	<0.001	460(3.4%)	117(3.9%)	0.170
Smoker	3910(28.7%)	1565(51.9%)	<0.001	2244(16.5%)	1224(40.6%)	<0.001
Number of Comorbidity (%)						
0	12.6	0.0	< 0.001	9.4	5.6	< 0.001
1	20.6	5.4		17.8	16.0	
2	21.1	15.5		19.8	20.8	
≥ 3	45.9	79.1		53.0	57.7	

#### Statistical analysis

**Fig. 2:**
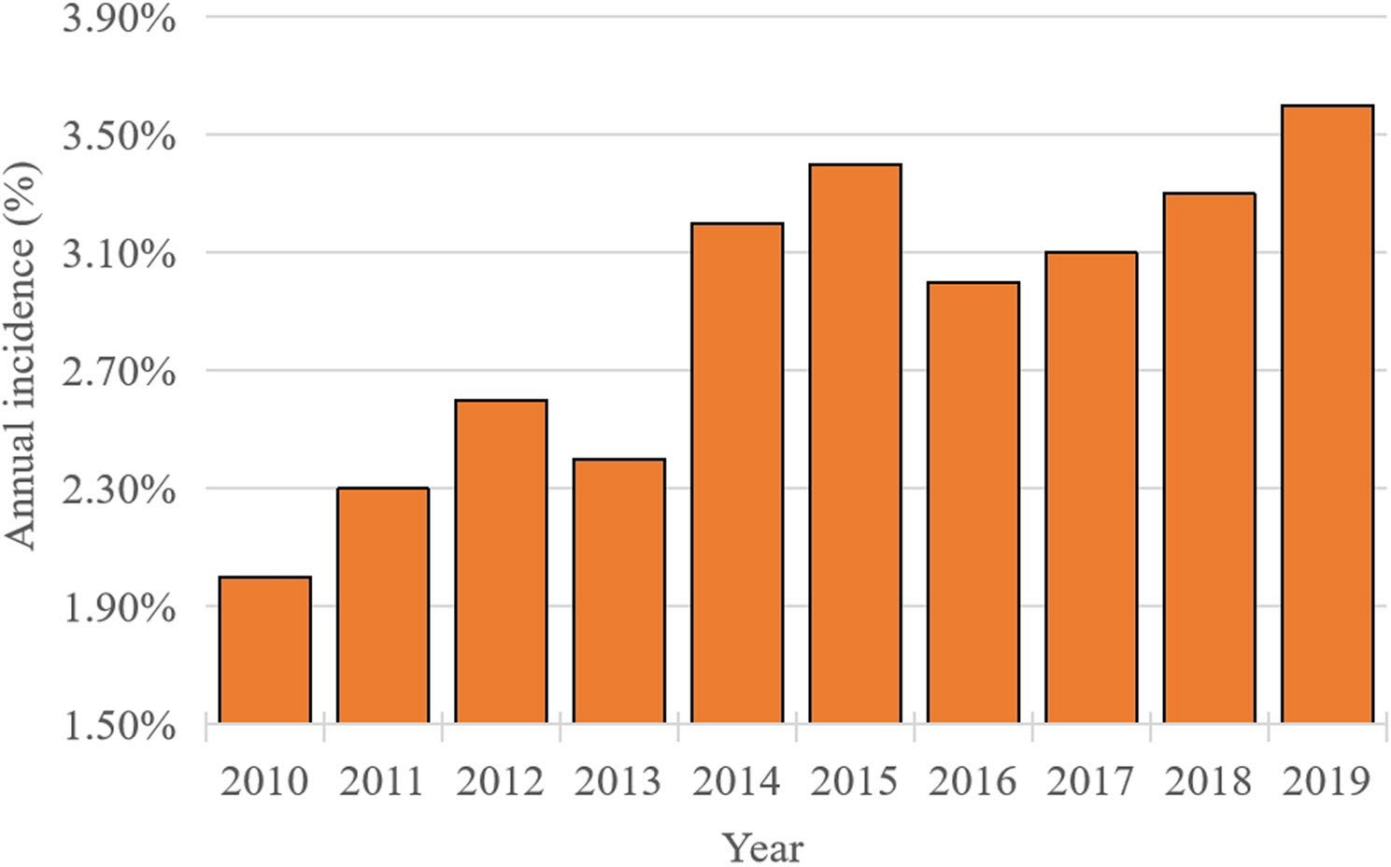
Temporal Trend in the Prevalence of AUD

We first determined the overall prevalence of AUD among patients undergoing catheter ablation between 2010 and 2019 (Fig. 2). To assess the independent association of AUD with post-procedural complications, we controlled for potential confounders, including age, demographics, hospital characteristics, and comorbidities. This process yielded 3,016 well-matched pairs. Balance was achieved, with all standardized mean differences below 0.10 (Table 2).

Continuous variables were analyzed using the Wilcoxon rank-sum test, and categorical variables were compared with Pearson’s chi-square test. We used univariate and multivariate logistic regression to assess the association between AUD and post-procedural complications. These models generated unadjusted (uOR) and adjusted odds ratios (aOR) with 95% confidence intervals (CIs). A two-sided *P*-value < 0.05 was considered statistically significant. All reported cost data were normalized to U.S. dollars (2019) using the consumer price index. Results are detailed in Table 3.

**Table 3:** Association between AUD and post-procedural complications: univariate and multivariable analysis

Perioperative complication	Before matching						After matching					
	non-AUD	AUD	*p*	Unadjusted OR (uOR)	95%CI	*p*	non-AUD	AUD	*p*	Adjusted OR (aOR)	95%CI	*p*
Cardiac complications	47,201 (44.5%)	1683 (54.1%)	< 0.001	1.14	1.05–1.22	0.001	7065 (51.8%)	1626 (53.9%)	0.038	1.03	0.95–1.11	0.510
Myocardial infarction	3375(3.2%)	163(5.2%)	< 0.001	1.20	1.02–1.41	0.031	625(4.6%)	155(5.1%)	0.193	0.99	0.82–1.19	0.926
Atrioventricular block	19,405(18.3%)	616(19.8%)	0.033	0.97	0.88–1.06	0.449	2807(20.6%)	603(20.0%)	0.460	0.91	0.82–1.00.82.00	0.060
Cardiac arrest	945(0.9%)	42(1.3%)	0.008	1.20	0.88–1.65	0.253	191(1.4%)	40(1.3%)	0.750	0.95	0.67–1.36	0.812
Heart failure	33,248(31.3%)	1246(40.0%)	< 0.001	1.16	1.07–1.25	< 0.001	6078(44.6%)	1436(47.6%)	0.003	1.08	0.99–1.17	0.051
Myocardial ischemia	1429(1.9%)	84(2.7%)	< 0.001	1.38	1.10–1.73	0.006	279(2.0%)	81(2.7%)	0.029	1.20	0.92–1.55	0.175
Acute myocardial infarction	4390(4.1%)	187(6.0%)	< 0.001	1.17	1.01–1.37	0.042	759(5.6%)	178(5.9%)	0.472	0.97	0.82–1.16	0.747
Pericardial complications	4723(4.5%)	95(3.1%)	< 0.001									
Acute pericarditis	550(0.5%)	7(0.2%)	0.023				69(0.5%)	7(0.2%)	0.043			
Hemopericardium	349(0.3%)	6(0.2%)	0.188				40(0.3%)	6(0.2%)	0.371			
Cardiac tamponade	1213(1.1%)	20(0.6%)	0.009				138(1.0%)	20(0.7%)	0.073			
Vascular complications	230 (0.2%)	5 (0.2%)	0.505	0.75	0.31–1.83	0.530						
Arteriovenous fistula	104(0.1%)	1(0.0%)	0.381	0.36	0.05–2.59	0.358	8(0.1%)	1(0.0%)	0.910	0.66	0.08–5.41	0.695
Vascular damage	4(0.0%)	0(0.0%)	1.000				2(0.0%)	0(0.0%)	1.000			
Retroperitoneal injury	23(0.0%)	1(0.0%)	0.500	1.01	0.13–7.66	0.994	6(0.0%)	1(0.0%)	1.000	0.54	0.06–4.88	0.588
Heart damage	36(0.0%)	1(0.0%)	1.000	0.89	0.12–6.60	0.907	7(0.1%)	1(0.0%)	1.000	0.50	0.06–4.23	0.523
Vascular complications of surgery	72(0.1%)	2(0.1%)	1.000	1.10	0.27–4.53	0.895	5(0.0%)	2(0.1%)	0.820	2.55	0.49–13.25	0.263
Pulmonary complications	7911 (7.5%)	354 (11.4%)	< 0.001	1.24	1.11–1.40	< 0.001	1468 (10.8%)	338 (11.2%)	0.485	0.92	0.80–1.04	0.185
Pneumothorax and hemothorax	395(0.4%)	11(0.4%)	0.864	0.97	0.53–1.78	0.932	61(0.4%)	10(0.3%)	0.377	0.72	0.36–1.43	0.347
Postoperative respiratory failure	7346(6.9%)	331(10.6%)	< 0.001	1.23	1.09–1.39	0.001	1394(10.2%)	316(10.5%)	0.681	0.89	0.78–1.02	0.099
Septal muscle paralysis and Phrenic nerve injury	145(0.1%)	8(0.3%)	0.127	2.03	0.99–4.20	0.055	21(0.2%)	8(0.3%)	0.185	1.92	0.83–4.44	0.128
Pulmonary embolism	463(0.4%)	21(0.7%)	0.049	1.12	0.72–1.75	0.615	97(0.7%)	20(0.7%)	0.773	0.85	0.51–1.40	0.530
Acute respiratory failure	5233(4.9%)	260(8.4%)	<0.001	1.35	1.18–1.55	< 0.001	976(7.2%)	248(8.2%)	0.043	1.03	0.88–1.20	0.671
Pneumonia	3959(3.7%)	178(5.7%)	< 0.001	1.21	1.03–1.42	0.018	746(5.5%)	172(5.7%)	0.616	0.91	0.76–1.08	0.290
Gastrointestinal complications	895 (0.8%)	46 (1.5%)	< 0.001	1.60	1.18–2.16	0.002	130(1.0%)	42 (1.4%)	0.031	1.37	0.95–1.97	0.088
Esophagitis	283(0.3%)	24(0.8%)	< 0.001	2.59	1.69–3.96	< 0.001	52(0.4%)	22(0.7%)	0.009	1.59	0.94–2.68	0.081
Esophageal ulcers	101(0.1%)	12(0.4%)	< 0.001	4.22	2.28–7.81	< 0.001	18(0.1%)	11(0.4%)	0.006	2.46	1.12–5.39	0.025
Esophageal strictures	229(0.2%)	8(0.3%)	0.626	1.20	0.59–2.45	0.618	25(0.2%)	8(0.3%)	0.360	1.39	0.61–3.17	0.436
Esophageal perforation	5(0.0%)	0(0.0%)	1.000				1(0.0%)	0(0.0%)	1.000			
Gastro-esophageal laceration-hemorrhage syndrome	18(0.0%)	1(0.0%)	0.423	2.06	0.27–15.94	0.489	1(0.0%)	1(0.0%)	0.330	3.80	0.23–63.64	0.353
Gastroparesis	313(0.3%)	4(0.1%)	0.089	0.35	0.13–0.93	0.035	43(0.3%)	3(0.1%)	0.041	0.34	0.10–1.13	0.078
Gastrointestinal bleeding	639(0.6%)	33(1.1%)	0.001	1.49	1.04–2.13	0.030	95(0.7%)	33(1.1%)	0.024	1.48	0.97–2.23	0.065
Genitourinary disease	16,923(15.9%)	649(20.8%)	< 0.001	1.03	0.94–1.13	0.496	2737(20.1%)	616(20.4%)	0.669	0.98	0.88–1.09	0.741
Acute kidney injury	13,418(12.6%)	568(18.2%)	< 0.001	1.12	1.01–1.23	0.030	2299(16.9%)	537(17.8%)	0.214	1.02	0.91–1.13	0.749
Inflammatory diseases of the central nervous system	26(0.0%)	3(0.1%)	0.049	2.16	0.62–7.51	0.227	9(0.1%)	3(0.1%)	0.807	1.10	0.28–4.36	0.896
Convulsion	419(0.4%)	26(0.8%)	< 0.001	1.84	1.23–2.76	0.003	76(0.6%)	24(0.8%)	0.126	1.36	0.85–2.19	0.202
Postoperative delirium	775(0.7%)	47(1.5%)	< 0.001	1.89	1.40–2.55	< 0.001	143(1.0%)	46(1.5%)	0.026	1.38	0.97–1.94	0.071
Stroke	1952 (1.8%)	59(1.9%)	0.820	1.08	0.83–1.41	0.559	242 (1.8%)	59(2.0%)	0.500	1.19	0.88–1.60	0.249
Embolism	1496 (1.4%)	57 (1.8%)	0.050	1.01	0.77–1.32	0.956	268 (2.0%)	54 (1.8%)	0.526	0.92	0.68–1.25	0.589
Postoperative shock	350 (0.3%)	11(0.4%)	0.822	1.08	0.59–1.98	0.809	65(0.5%)	10(0.3%)	0.281	0.80	0.40–1.57	0.512
Blood transfusion	3932 (3.7%)	117 (3.8%)	0.878	0.94	0.78–1.14	0.513	584(4.3%)	111(3.7%)	0.133	0.92	0.74–1.14	0.460
Septicemia	1904(1.8%)	99(3.2%)	< 0.001	1.29	1.05–1.59	0.017	415(3.0%)	91(3.0%)	0.937	0.87	0.70–1.14	0.374
Chest pain	2636(2.5%)	101(3.2%)	0.007	1.02	0.83–1.25	0.843	393(2.9%)	98(3.2%)	0.282	0.97	0.77–1.23	0.815
Electrolyte imbalance	15,028(14.2%)	773(24.8%)	< 0.001	1.47	1.35–1.61	<0.001	2988(21.9%)	719(23.8%)	0.022	1.02	0.92–1.12	0.748
Severe malnutrition	2661(2.5%)	130(4.2%)	< 0.001	1.36	1.13–1.63	0.001	470(3.4%)	120(4.0%)	0.154	1.11	0.90–1.37	0.331
Acute respiratory distress syndrome	4363(4.1%)	198(6.4%)	< 0.001	1.31	1.13–1.53	< 0.001	767(5.6%)	192(6.4%)	0.115	1.04	0.88–1.24	0.616
Continuous trauma ventilation	3135(3.0%)	137(4.4%)	< 0.001	1.21	1.01–1.45	0.038	539(4.0%)	133(4.4%)	0.250	1.04	0.85–1.27	0.704
Thrombocytopenia	3694(3.5%)	263(8.4%)	< 0.001	2.07	1.80–2.36	< 0.001	962(7.1%)	238(7.9%)	0.109	1.04	0.89–1.22	0.605
Liver disease	81(0.1%)	10(0.3%)	< 0.001	3.13	1.57–6.24	0.001	24(0.2%)	10(0.3%)	0.087	1.56	0.72–3.38	0.263
Hemorrhage/Seroma/Hematoma	2308(2.2%)	43(1.4%)	0.003	0.71	0.52–0.96	0.028	262(1.9%)	42(1.4%)	0.049	0.79	0.56–1.10	0.158

*OR* Odds ratio, *CI* Confidence interval

To investigate the apparent survival advantage in the AUD cohort, we first performed a pre-specified subgroup analysis of in-hospital mortality by admission type (emergency vs. non-emergency; Table 4). To further explore this finding, we conducted a post-hoc sensitivity analysis of all recorded in-hospital deaths (*n* = 212) from the matched cohort. Among the patients who died, we compared demographics, hospital characteristics, and comorbidities between those with and without AUD (Table 5).

**Table 4:** In-hospital outcomes were compared by the presence of patients with AUD after arrhythmia ablation

Outcome	Before matching			After matching		
	non-AUD	AUD	*p* value	non-AUD	AUD	*p* value
In-hospital mortality	891(0.9%)	18(0.6%)	0.045	194(1.4%)	18(0.6%)	< 0.001
In-hospital mortality by admission type						
Emergency admissions				164/10,732 (1.5%)	17/2401 (0.7%)	0.002
Non-emergency admissions				30/2899 (1.0%)	1/615 (0.2%)	0.036
Total complications	66,479(62.6%)	2422(77.8%)	< 0.001	9759(71.6%)	2334(77.4%)	< 0.001
LOS (median, d)	3.0 (2.0–6.0)	4.0 (3.0–8.0)	< 0.001	4.0 (2.0–7.0)	4.0 (2.0–8.0)	< 0.001
TOTCHG (median, $)	101994.0 (66902.0–155382.5.0.5)	110971.0 (72216.0–172424.0.0.0)	< 0.001	116503.00 (77968.65–181360.00.65.00)	118427.97 (77900.67–183408.72.67.72)	< 0.001

*LOS* Length of stay, *TOTCHE* Total charge

**Table 5:** Baseline characteristics and comorbidities of deceased patients stratified by AUD status

Demographic/comorbidity	After matching		
	non-AUD	AUD	*p* value
Age group (%)			
18–44	30.4	27.8	0.440
45–64	27.8	44.4	
65–74	27.3	22.2	
≥ 75	14.4	5.6	
Gender (%)			
Male	87.1	100	0.105
Female	12.9	0.0	
Race (%)			
White	72.2	72.2	0.606
Black	16.0	5.6	
Hispanic	4.1	5.6	
Asian or Pacific Islander	0.5	0.0	
Native American	0.5	0.0	
Other	6.7	16.7	
Type of insure (%)			
Medicare	59.3	61.1	0.568
Medicaid	13.4	11.1	
Private insurance	23.7	22.2	
Self-pay	1.0	5.6	
Other	2.6	0.0	
Bed size of hospital (%)			
Small	9.3	11.1	0.954
Medium	15.5	16.7	
Large	75.3	72.2	
Elective admission (%)	15.5	5.6	0.255
Type of hospital (teaching %)	82.5	100.0	0.053
Location of hospital (urban, %)	97.4	100.0	0.491
Preoperative comorbidities			
Deficiency anemia	31 (16.0%)	5 (27.8%)	0.202
Rheumatoid arthritis/collagen vascular diseases	3(1.5%)	0(0.0%)	0.595
Chronic blood loss anemia	2 (1.0%)	0 (0.0%)	0.665
Congestive heart failure	101(52.1%)	10(55.6%)	0.776
Chronic pulmonary disease	69(35.6%)	8(44.4%)	0.454
Coagulopathy	81(41.8%)	8(44.4%)	0.825
Depression	23 (11.9%)	0 (0.0%)	0.230
Diabetes, uncomplicated	29(14.9%)	4 (22.2%)	0.415
Diabetes with chronic complications	23(11.9%)	2 (11.1%)	0.925
Drug abuse	3(1.5%)	4(22.2%)	<0.001
Hypertension	130 (67.0%)	9 (50.0%)	0.146
Hypothyroidism	12(6.2%)	2 (11.1%)	0.421
Liver disease	37(19.1%)	4(22.2%)	0.746
Lymphoma	1(0.5%)	0(0.0%)	0.760
Fluid and electrolyte disorders	147(75.8%)	13 (72.2%)	0.738
Metastatic cancer	9(4.6%)	1(5.6%)	0.861
Other neurological disorders	46(23.7%)	5(27.8%)	0.699
Obesity	37(19.1%)	6 (33.3%)	0.150
Paralysis	5 (2.6%)	0 (0.0%)	0.491
Peripheral vascular disorders	26(13.4%)	2 (11.1%)	0.784
Psychoses	5(2.6%)	1 (5.6%)	0.466
Pulmonary circulation disorders	31(16.0%)	0 (0.0%)	0.066
Renal failure	70 (36.1%)	10 (55.6%)	0.103
Solid tumor without metastasis	9(4.6%)	0(0.0%)	0.350
Peptic ulcer disease excluding bleeding	1(0.5%)	0(0.0%)	0.760
Valvular disease	28 (14.4%)	2 (11.1%)	0.699
Weight loss	49(25.3%)	3(16.7%)	0.418
Smoker	18(9.3%)	3(16.7%)	0.316
Number of Comorbidity (%)			
0	1.0	0.0	0.840
1	2.6	5.6	
2	8.8	11.1	
≥ 3	87.6	83.3	

## Results

### Baseline characteristics

From 2010 to 2019, the annual incidence of AUD among patients undergoing catheter ablation increased steadily (Fig. 2). Within the total cohort of 109,226 patients, 3,113 (2.85%) had a documented diagnosis of AUD. Prior to PSM, we found that patients with AUD were significantly younger (median age 60.0 vs. 67.0 years, *P* < 0.001) and more likely to be male (86.9% vs. 60.6%, *P* < 0.001). Following 1:5 PSM, the final analysis included 3,016 patients with AUD and 13,631 matched comparators without AUD. The matched cohorts were well-balanced for all baseline characteristics, as detailed in Table 2.

### Hospital care characteristics

Prior to matching, patients with AUD had a longer median length of stay (4.0 days vs. 3.0 days, *P* < 0.001) and incurred higher total hospitalization charges ($118,427 vs. $116,503, *P* < 0.001) (Table 4). Insurance coverage also differed significantly between the cohorts (*P* < 0.001). Specifically, patients with AUD had a lower rate of Medicare coverage (40.4% vs. 58.9%) but a higher rate of Medicaid coverage (17.5% vs. 6.1%) compared to non-AUD patients.

### Comorbidity profile

Before PSM, patients with AUD had a significantly higher burden of comorbidities, including congestive heart failure (26.7% vs. 19.4%), chronic pulmonary disease (31.8% vs. 22.0%), coagulopathy (11.5% vs. 4.7%), and drug abuse (11.9% vs. 1.2%) (all *P* < 0.001). After matching, significant disparities persisted, with the AUD cohort demonstrating markedly higher rates of drug abuse (10.3% vs. 5.5%), smoking (40.6% vs. 16.5%), and liver disease (11.3% vs. 7.8%) compared to non-AUD controls (all *P* < 0.001; Table 2).

### Risk factors for adverse outcomes

Multivariate logistic regression identified several factors associated with post-procedural complications in patients with AUD. Advanced age (≥ 65 years), female sex, Black or Native American race, a higher Charlson Comorbidity Index, and insurance status, including Medicaid, self-pay, or uninsured status, were significantly associated with an increased risk of complications. Conversely, elective admission and larger hospital bed size were protective factors (Table 6).

**Table 6:** Risk factors associated with AUD

Variable	Multivariate Logistic Regression		
	Unadjusted OR (uOR)	Wald 95%CI	*p* value
Age ≥ 65 years old	0.54	0.49–0.61	<0.001
Female	0.24	0.22–0.27	<0.001
Race			
White	Ref	——	——
Black	1.17	1.05–1.31	0.005
Hispanic	0.99	0.84–1.16	0.885
Asian or Pacific Islander	0.58	0.39–0.85	0.006
Native American	1.89	1.17–3.05	0.009
Other	0.95	0.83–1.09	0.444
Number of Comorbidity			
0	Ref	——	——
1	1.72	1.42–2.08	<0.001
2	2.36	1.93–2.88	<0.001
≥ 3	2.93	2.32–3.70	<0.001
Type of insurance			
Medicare	Ref	——	——
Medicaid	1.78	1.56–2.04	<0.001
Private insurance	1.04	0.92–1.16	0.545
Self-pay	2.55	2.14–3.04	<0.001
No charge	3.64	2.51–5.29	<0.001
Other	1.44	1.17–1.76	<0.001
Bed size of hospital			
Small	Ref	——	——
Medium	0.89	0.77–1.03	0.123
Large	0.83	0.73–0.95	0.006
Elective admission	0.47	0.43–0.52	<0.001
Teaching hospital	1.03	0.92–1.14	0.638
Urban hospital	1.03	0.79–1.36	0.818
Region of hospital			
Northeast	Ref	——	——
Midwest or North Central	1.19	1.06–1.33	0.003
South	1.05	0.95–1.17	0.320
West	1.17	1.03–1.33	0.014

*OR* Odds ratio, *CI* Confidence interval

### Postoperative complications

Following PSM and multivariate adjustment, patients with AUD demonstrated a significantly elevated risk of gastrointestinal complications compared to their non-AUD counterparts (1.4% vs. 1.0%, aOR: 1.44, 95% CI: 1.03–2.00, *P* = 0.031). This risk was particularly pronounced for esophageal ulcers (0.4% vs. 0.1%, aOR: 2.46, 95% CI: 1.12–5.39, *P* = 0.025). In contrast, the incidence of other postoperative complications, including cardiac, vascular, pulmonary, genitourinary, and infectious events, was comparable between the cohorts. Strikingly, despite the increased morbidity, the AUD cohort exhibited significantly lower in-hospital mortality (0.6% vs. 1.4%, *P* < 0.001) after matching (Table 4).

### Analysis of mortality in patients with AUD

Demographic and clinical differences were observed among patients who died. As detailed in Table 5, deceased patients in the AUD cohort were disproportionately male (100% vs. 87.1%, *P* = 0.105) and a greater proportion were aged 45–64 years (44.4% vs. 27.8%, *P* = 0.440), although these differences did not reach statistical significance. Critically, AUD decedents had a much higher prevalence of concomitant drug abuse (22.2% vs. 1.5%, *P* < 0.001). Conversely, they demonstrated a lower burden of several cardiovascular and metabolic comorbidities, including pulmonary circulation disorders (0.0% vs. 16.0%, *P* = 0.066) and uncomplicated hypertension (50.0% vs. 67.0%, *P* = 0.146). A non-significant trend also indicated a lower prevalence of depression (0.0% vs. 11.9%, *P* = 0.230). Furthermore, a smaller proportion of AUD decedents had three or more comorbid conditions (83.3% vs. 87.6%).

## Discussion

AUD constitutes a clinically significant comorbid condition in patients undergoing treatment for cardiac arrhythmias. Sustained heavy drinking drives disease progression through dual mechanisms of myocardial structural alteration and electrical remodeling, collectively enhancing susceptibility to arrhythmogenesis [5]. Examination of 109,226 catheter ablation procedures documented in the NIS database from 2010 to 2019 revealed 3,113 (2.85%) individuals with concurrent AUD. Throughout this decade, the annual prevalence of AUD within this clinical population showed a consistent upward trajectory (Fig. 2), a pattern that highlights the clinical imperative to delineate procedural outcomes in this expanding patient group [8]. Although catheter ablation represents a well-established intervention for rhythm disorders [9], our investigation reveals that AUD patients manifest a characteristic and heightened susceptibility to particular post-procedural complications, with gastrointestinal manifestations, including esophagitis and esophageal ulceration, being particularly prominent.

The significantly elevated esophageal ulcer incidence observed in our AUD cohort (0.4% vs. 0.1%, *P* = 0.006) represents a clinically significant finding. This complication likely results from synergistic effects between alcohol-induced esophageal vulnerability and ablation-related procedural stress. Chronic alcohol intake is known to compromise esophageal integrity via multiple mechanisms, including reducing lower esophageal sphincter pressure, enhancing gastric acid secretion, and diminishing protective salivary bicarbonate production [10]. Moreover, alcohol induces manometric abnormalities, including heightened esophageal contraction amplitude, potentially representing compensatory responses that nonetheless contribute to esophageal dysmotility and functional instability [11]. Over time, this predisposition can impair mucosal defenses. During catheter ablation, the delivery of thermal energy near the esophageal wall could therefore affect tissues that are already weakened [12]. The procedure may not necessarily create de novo injury but could rather induce or intensify chronic, subclinical esophagopathy, triggering increased clinical complications. This pathophysiological sequence emphasizes the necessity for improved preoperative risk stratification and deployment of customized protective protocols.

Appropriate preventive strategies include meticulous intraoperative esophageal monitoring, such as luminal temperature sensing, combined with optimized ablation parameters (reduced power and shorter duration) and, when feasible, the use of mechanical displacement devices to increase the distance between the atrium and esophagus [13]. These targeted measures are important for limiting the combined risk posed by chronic alcohol-related esophageal fragility and procedure-related thermal injury, ultimately improving the safety of catheter ablation in this vulnerable patient group.

Patients with AUD also experienced prolonged LOS and higher healthcare costs, driven primarily by a greater comorbidity burden and a higher incidence of post-procedural complications. Previous studies indicate that individuals with AUD often present with more complex clinical presentations, necessitating more intensive medical management and extended hospitalization [14]. The economic impact of catheter ablation in this population is substantial, reflecting not only the cost of specialized ablation equipment and institutional as well as professional fees but also the additional resources required to manage complications. As a result, the financial burden is disproportionately higher for patients with AUD compared with those without AUD. Addressing these challenges will require strategies that optimize patient selection, standardize clinical pathways, and incorporate cost-effective technologies. Such measures are crucial to help mitigate the economic impact on this vulnerable patient group [15].

Despite having higher complication rates and longer hospital stays, patients with AUD exhibited reduced in-hospital mortality, a paradoxical finding that warrants careful interpretation. This difference is likely explained by the younger age and distinct demographic characteristics of the AUD cohort undergoing ablation. Herein, patients with AUD were markedly younger than non-AUD counterparts, a disparity persisting after rigorous PSM (median age: 60.0 vs. 62.0 years, *P* < 0.001) (Table 2). This observation is consistent with established epidemiologic patterns showing that AUD is most prevalent in early to middle adulthood [16]. Consequently, AUD patients deemed eligible for catheter ablation inherently comprise a younger demographic possessing greater physiological reserve, potentially offsetting risks associated with their elevated comorbidity burden. Our sensitivity analysis of mortality cases (Table 5) further supports this trend, revealing that AUD decedents concentrated more frequently in younger age brackets relative to non-AUD decedents. Thus, the observed survival advantage likely represents a reflection of selection bias rather than any protective biological effect of AUD. AUD patients who undergo ablation appear to represent a younger and potentially more resilient subset of the broader AUD population, a pattern that has also been reported in other clinical settings involving hospitalized patients with substance use disorders [17, 18].

Our pre-specified subgroup analysis of in-hospital mortality by admission type provides compelling evidence for selection bias. The survival advantage associated with AUD was most pronounced in the non-emergency admission subgroup, where mortality was 80% lower in AUD patients (0.2% vs. 1.0%; *P* = 0.036), compared with a 53% reduction in the emergency subgroup (0.7% vs. 1.5%; *P* = 0.002) (Table 4). This strongly indicates that AUD patients selected for elective ablation constitute a distinct, clini

cally more resilient subgroup. This finding aligns with established clinical practice, wherein elective procedural selection inherently filters out individuals with severe, decompensated AUD, yielding a cohort of “healthier survivors” within the broader AUD population [19]. Our sensitivity analysis of decedents (Table 5) further substantiates this interpretation of divergent risk profiles. Among deceased patients, those with AUD were younger and demonstrated a distinct comorbidity profile, marked by higher rates of concomitant drug abuse but lower prevalence of chronic cardiopulmonary disease. These findings suggest that the pathways to in-hospital death differ between cohorts. In non-AUD patients, mortality appears to be driven predominantly by advanced age and progressive conditions like congestive heart failure (CHF), a well-established predictor of early post-ablation mortality in patients with AF [20], highlighting the adverse interplay between heart failure and arrhythmia outcomes [21]. Conversely, mortality in this selected AUD cohort was more commonly associated with acute events linked to their specific risk profile, including complications related to polysubstance use.

The structured, protocol-driven environment of an inpatient hospitalization may mitigate the acute risks prevalent in this population. Standardized care pathways are consistently associated with improved postoperative recovery and reduced complication rates [22]. For this selected AUD cohort, the implementation of comprehensive preoperative assessments and dedicated perioperative protocols, as recommended for other high-risk surgical populations [19, 23], could help optimize in-hospital management, thereby attenuating immediate risks and potentially contributing to the observed short-term mortality advantage. Despite extensive sensitivity analyses, including an assessment of sex disparities (Table 7), we found no additional compelling explanations for the mortality difference within the available data. Furthermore, although some studies suggest light-to-moderate alcohol consumption may have conferred cardioprotective benefits [24–26], these findings are unlikely to be applicable to a clinical AUD population undergoing catheter ablation. Consequently, the lower in-hospital mortality observed in the AUD group should not be interpreted as an indicator of improved long-term prognosis, but rather as a context-specific outcome driven by protocolized inpatient care.

**Table 7:** Characteristics and outcomes of female ablation Patients, by AUD status

Demographic/comorbidity/ Perioperative complication	After matching		
	non-AUD	AUD	*p* value
Total (n = count)	1796	404	
Age group (%)			
18–44	41.1	44.6	0.001
45–64	23.8	28.2	
65–74	22.0	21.3	
≥ 75	13.0	5.9	
Race (%)			
White	65.5	68.6	0.338
Black	17.4	17.8	
Hispanic	7.3	5.0	
Asian or Pacific Islander	1.1	0.5	
Native American	0.9	1.5	
Other	7.7	6.7	
Type of insure (%)			
Medicare	48.8	47.3	0.030
Medicaid	17.5	19.8	
Private insurance	24.7	21.3	
Self-pay	6.5	6.4	
No charge	1.0	1.2	
Other	1.6	4.0	
Bed size of hospital (%)			
Small	8.8	11.1	0.322
Medium	21.6	21.8	
Large	69.6	67.1	
Region of hospital(%)			
Northeast	20.2	18.1	0.350
Midwest or North Central	22.0	20.8	
South	40.3	40.1	
West	17.5	21.0	
Elective admission (%)	19.4	23.3	0.082
Type of hospital (teaching %)	79.2	80.4	0.569
Location of hospital (urban, %)	97.8	98.8	0.204
Preoperative comorbidities			
Acquired immune deficiency syndrome	8(0.4%)	2 (0.5%)	0.893
Deficiency anemia	231(12.9%)	44(10.9%)	0.279
Rheumatoid arthritis/collagen vascular diseases	77(4.3%)	10(2.5%)	0.091
Chronic blood loss anemia	18(1.0%)	3(0.7%)	0.628
Congestive heart failure	455(25.3%)	92(22.8%)	0.282
Chronic pulmonary disease	671(37.4%)	156(38.6%)	0.638
Coagulopathy	165(9.2%)	41(10.1%)	0.549
Depression	300(16.7%)	70(17.3%)	0.762
Diabetes, uncomplicated	237(13.2%)	57(14.1%)	0.626
Diabetes with chronic complications	113(6.3%)	19(4.7%)	0.224
Drug abuse	157(8.7%)	40(9.9%)	0.461
Hypertension	1218(67.8%)	267(66.1%)	0.503
Hypothyroidism	274(15.3%)	62(15.3%)	0.964
Liver disease	171(9.5%)	45(11.1%)	0.324
Fluid and electrolyte disorders	533(29.7%)	144(35.6%)	0.019
Other neurological disorders	105(5.8%)	28(6.9%)	0.409
Obesity	427(23.8%)	71(17.6%)	0.007
Paralysis	13(0.7%)	3(0.7%)	0.968
Peripheral vascular disorders	102(5.7%)	24(5.9%)	0.838
Psychoses	121(6.7%)	36(8.9%)	0.125
Pulmonary circulation disorders	114(6.3%)	32(7.9%)	0.251
Renal failure	183(10.2%)	43(10.6%)	0.786
Peptic ulcer disease excluding bleeding	13(0.7%)	2(0.5%)	0.614
Valvular disease	216(12.0%)	39(9.7%)	0.178
Weight loss	63(3.5%)	18(4.5%)	0.361
Number of Comorbidity (%)			
0	8.9	4.7	0.044
1	13.8	13.9	
2	18.2	17.8	
≥ 3	59.2	63.6	
Died (%)	1.4	0.0	0.017
Perioperative complication			
Cardiac complications	867(48.3%)	189(46.8%)	0.588
Myocardial infarction	64(3.6%)	13(3.2%)	0.733
Atrioventricular block	355(19.8%)	80(19.8%)	0.987
Cardiac arrest	22(1.2%)	7(1.7%)	0.419
Heart failure	753(41.9%)	159(39.4%)	0.343
Myocardial ischemia	38(2.1%)	10(2.5%)	0.655
Acute myocardial infarction	84(4.7%)	17(4.2%)	0.684
Pericardial complications			
Acute pericarditis	6(0.3%)	0(0.0%)	0.245
Hemopericardium	5(0.3%)	2(0.5%)	0.485
Cardiac tamponade	21(1.2%)	3(0.7%)	0.456
Vascular complications			
Retroperitoneal injury	1(0.1%)	0(0.0%)	0.635
Vascular complications of surgery	0(0.0%)	1(0.2%)	0.184
Pulmonary complications	200(11.1%)	48(11.9%)	0.669
Pneumothorax and hemothorax	6(0.3%)	1(0.2%)	0.780
Postoperative respiratory failure	191(10.6%)	43(10.6%)	0.996
Septal muscle paralysis and Phrenic nerve injury	3(0.2%)	2(0.5%)	0.211
Pulmonary embolism	13(0.7%)	2(0.5%)	0.865
Acute respiratory failure	122(6.8%)	32(7.9%)	0.422
Pneumonia	110(6.1%)	21(5.2%)	0.477
Gastrointestinal complications	30(1.7%)	6(1.5%)	0.791
Esophagitis	11(0.6%)	2(0.5%)	0.781
Esophageal ulcers	5(0.3%)	2(0.5%)	0.485
Esophageal strictures	6(0.3%)	2(0.5%)	0.627
Gastroparesis	12(0.7%)	0(0.0%)	0.099
Gastrointestinal bleeding	15(0.8%)	5(1.2%)	0.631
Genitourinary disease	330(18.4%)	79(19.6%)	0.582
Acute kidney injury	239(13.3%)	61(15.1%)	0.343
Inflammatory diseases of the central nervous system	1(0.1%)	0(0.0%)	1.000
Convulsion	11(0.6%)	2(0.5%)	1.000
Postoperative delirium	17(0.9%)	9(2.2%)	0.041
Stroke	37(2.1%)	10(2.5%)	0.602
Embolism	33(1.8%)	4(1.0%)	0.231
Postoperative shock	11(0.6%)	1(0.2%)	0.368
Blood transfusion	104(5.8%)	21(5.2%)	0.642
Septicemia	52(2.9%)	11(2.7%)	0.851
Chest pain	60(3.3%)	10(2.5%)	0.370
Electrolyte imbalance	464(25.8%)	131(32.4%)	0.007
Severe malnutrition	71(4.0%)	21(5.2%)	0.259
Acute respiratory distress syndrome	92(5.1%)	27(6.7%)	0.210
Continuous trauma ventilation	61(3.4%)	15(3.7%)	0.753
Thrombocytopenia	123(6.8%)	29(7.2%)	0.813
Liver disease	3(0.2%)	1(0.2%)	0.556
Hemorrhage/Seroma/Hematoma	41(2.3%)	7(1.7%)	0.494

The peri-procedural period of catheter ablation represents a critical window for therapeutic intervention. Evidence demonstrates that preoperative alcohol cessation programs are both feasible and effective in reducing postoperative complications while also promoting sustained abstinence [27]. This is particularly important given the well-established, dose-dependent relationship between alcohol consumption and AF recurrence, in which even moderate intake markedly increases the risk of post-ablation recurrence [28]. The underlying pathophysiology likely reflects alcohol-induced autonomic imbalance, electrophysiologic instability, and structural myocardial remodeling, which together promote a pro-arrhythmic substrate [1]. Accordingly, clinical studies consistently demonstrate higher procedural success rates among abstainers relative to active drinkers [21]. Although our study assessed in-hospital mortality rather than long-term outcomes, diminished arrhythmia recurrence serves as a key determinant of long-term survival by lowering the risk of subsequent hospitalization for heart failure and thromboembolic events. Thus, a perioperative focus on alcohol cessation in this high-risk AUD cohort may have attenuated their immediate post-procedural arrhythmic risk, potentially contributing to the observed short-term mortality advantage.

Therefore, a key clinical strategy is the implementation of a structured, stepped-care model for alcohol cessation, initiated well before ablation. This approach begins with systematic preoperative screening for AUD, followed by risk-stratified interventions. Patients with hazardous drinking patterns may benefit from Alcohol Brief Interventions (ABIs) delivered by the cardiac team to motivate alcohol reduction. Those with mild to moderate alcohol dependence should be referred for specialized psychological interventions, such as cognitive-behavioral therapy. For patients with established alcohol dependence, a supervised withdrawal protocol, conducted in an outpatient or inpatient setting according to severity, is an essential component of pre-procedural optimization, analogous to standard anticoagulation management [29]. Systematic reviews show that intensive alcohol cessation programs delivered over four to eight weeks preoperatively can significantly reduce both complication rates and postoperative alcohol consumption. The physiological rationale for this approach is especially robust. Notably, abstinence promotes the reversal of alcohol-induced autonomic dysfunction and adverse electrical remodeling, both of which influence arrhythmia recurrence and periprocedural instability [23, 30]. Emerging evidence indicates that alcohol reduction, particularly in heavy drinkers, is associated with a substantially lower rate of arrhythmia recurrence post-ablation, with benefits manifesting shortly after the procedure [31]. Adherence can be objectively monitored through random testing for metabolites such as ethyl glucuronide [29]. In addition, a multidisciplinary model involving electrophysiologists, addiction specialists, and mental health professionals is critical for optimal patient management.

Nevertheless, our findings should be interpreted within the context of several limitations inherent to analyses of administrative databases. First, reliance on ICD codes for identifying AUD and complications introduces potential misclassification bias, as coding practices vary across institutions and may fail to capture subtle clinical details [32, 33]. The documented moderate reliability (kappa of approximately 0.72) and differential performance (sensitivity 95% vs. specificity 77%) of AUD codes suggest our cohort may not fully represent the complete spectrum of alcohol-related disorders in this population [34]. Second, the cross-sectional nature of the NIS database, which captures hospitalization events rather than longitudinal patient trajectories, precludes analysis of critical long-term outcomes. Consequently, we were unable to assess arrhythmia recurrence, long-term mortality, and other endpoints that manifest after discharge, a recognized limitation of NIS-based research [35, 36]. Thirdly, although we used rigorous propensity score matching to balance measured covariates, the absence of detailed clinical data, including exact alcohol consumption levels, medication adherence, and laboratory values, means that residual confounding by unmeasured factors may persist.

Moreover, the exclusive use of inpatient data fundamentally limited the interpretation and generalizability of our findings. Although we documented higher in-hospital complication rates in patients with AUD, the absence of long-term follow-up precludes a comprehensive assessment of the procedure’s therapeutic success in this population. This represents a critical limitation, as established evidence demonstrates that alcohol cessation significantly reduces AF recurrence risk post-ablation, suggesting that the benefits of perioperative management extend far beyond the initial hospitalization [27, 28]. Furthermore, we cannot determine whether the observed increase in gastrointestinal complications leads to long-term sequelae or compromises quality of life. Therefore, future research must employ prospective designs that incorporate standardized AUD assessments, detailed consumption metrics, and systematic longitudinal follow-up. These studies are essential to validate our findings and definitively elucidate the relationship between perioperative alcohol interventions and sustained arrhythmia-free survival.

## Conclusion

In our national analysis of 109,226 catheter ablation procedures, patients with AUD, representing 2.85% of the cohort, exhibited distinct clinical profiles and outcomes. This group was characterized by a younger age, male predominance, and carried a higher burden of certain comorbidities, including drug abuse, smoking, and liver disease. Despite adjustment, the AUD cohort experienced longer hospitalizations, higher costs, and a significantly elevated risk of gastrointestinal complications, yet paradoxically showed lower in-hospital mortality. Together, these findings highlight the need for targeted preoperative optimization and tailored perioperative management for patients with AUD undergoing catheter ablation. Therefore, future research should develop and validate interventions to reduce these procedure-specific risks and improve outcomes in this vulnerable population.

## Supplementary Information

Supplementary Material 1.

## Data Availability

The datasets generated and/or analyzed during the current study are available in the NIS repository, https://www.hcup-us.ahrq.gov/nisoverview.jsp. The analyzed datasets are also available from the corresponding author on reasonable request.

## Abbreviations

AUD: Alcohol Use Disorder
AF: Atrial Fibrillation
VT: Ventricular Tachycardia
NIS: Nationwide Inpatient Sample
HCUP: Healthcare Cost and Utilization Project
AHRQ: Agency for Healthcare Research and Quality
ICD: International Classification of Diseases
PSM: Propensity Score Matching
LOS: Length of Stay
uOR: unadjusted Odds Ratio
aOR: adjusted Odds Ratio
CI: Confidence Interval

## References

[1] Wong CX, Tu SJ, Marcus GM. Alcohol and arrhythmias. JACC Clin Electrophysiol. 2023;9(2):266–79. 10.1016/j.jacep.2022.10.023. 36858701 10.1016/j.jacep.2022.10.023

[2] Brunner S, Krewitz C, Winter R, et al. Acute alcohol consumption and arrhythmias in young adults: the MunichBREW II study. Eur Heart J. 2024;45(46):4938–49. 10.1093/eurheartj/ehae695. 39363568 10.1093/eurheartj/ehae695

[3] Guzzo-Merello G, Dominguez F, González-López E, et al. Malignant ventricular arrhythmias in alcoholic cardiomyopathy. Int J Cardiol. 2015;199:99–105. 10.1016/j.ijcard.2015.07.029. 26188828 10.1016/j.ijcard.2015.07.029

[4] Hosseini SM, Rozen G, Saleh A, et al. Catheter ablation for cardiac arrhythmias: utilization and in-hospital complications, 2000 to 2013. JACC Clin Electrophysiol. 2017;3(11):1240–8. 10.1016/j.jacep.2017.05.005. 29759619 10.1016/j.jacep.2017.05.005

[5] Qiao Y, Shi R, Hou B, et al. Impact of alcohol consumption on substrate remodeling and ablation outcome of paroxysmal atrial fibrillation. J Am Heart Assoc. 2015;4(11):e002349. 10.1161/JAHA.115.002349. 26553213 10.1161/JAHA.115.002349 PMC4845226

[6] Mao X, Liang C, Li X, et al. The impact of long-term aspirin use on the patients undergoing shoulder arthroplasty. J Orthop Surg Res. 2023;18(1):894. 10.1186/s13018-023-04374-4. Published 2023 Nov 23. 37993872 10.1186/s13018-023-04374-4 PMC10666390

[7] Li X, Xie H, Liu S, et al. Analysis of the incidence and risk factors of blood transfusion in total knee revision: a retrospective nationwide inpatient sample database study. BMC Musculoskelet Disord. 2024;25(1):225. 10.1186/s12891-024-07331-2. 38509493 10.1186/s12891-024-07331-2 PMC10953239

[8] Bukata IT, Tegene E, Gobena T, Woldesenbet YM. Prevalence and determinants of cardiac arrhythmias and conduction anomalies in adults aged ≥ 40 years in Jimma Town, Southwest of Ethiopia: a cross-sectional study. Afr Health Sci. 2022;22(2):236–46. 10.4314/ahs.v22i2.27. 36407368 10.4314/ahs.v22i2.27 PMC9652622

[9] Stevenson WG, Tedrow UB, Reddy V, et al. Infusion needle radiofrequency ablation for treatment of refractory ventricular arrhythmias. J Am Coll Cardiol. 2019;73(12):1413–25. 10.1016/j.jacc.2018.12.070. 30922472 10.1016/j.jacc.2018.12.070

[10] Chang CH, Wu CP, Wang JD, et al. Alcohol and tea consumption are associated with asymptomatic erosive esophagitis in Taiwanese men. PLoS One. 2017;12(3):e0173230. 10.1371/journal.pone.0173230. (**Published 2017 Mar 6**). 28264069 10.1371/journal.pone.0173230 PMC5338804

[11] Haber PS, Kortt NC. Alcohol use disorder and the gut. Addiction. 2021;116(3):658–67. 10.1111/add.15147. 32511812 10.1111/add.15147

[12] Kaneshiro T, Takeishi Y. Esophageal thermal injury in catheter ablation of atrial fibrillation. Fukushima J Med Sci. 2021;67(3):95–101. 10.5387/fms.2021-23. 34803083 10.5387/fms.2021-23 PMC8784191

[13] Cronin EM, Bogun FM, Maury P, et al. 2019 HRS/EHRA/APHRS/LAHRS expert consensus statement on catheter ablation of ventricular arrhythmias. Europace. 2019;21(8):1143–4. 10.1093/europace/euz132. 31075787 10.1093/europace/euz132 PMC7967791

[14] Schwarzinger M, Thiébaut S, Baillot S, Mallet V, Rehm J. Alcohol use disorders and associated chronic disease - a national retrospective cohort study from France. BMC Public Health. 2017;18(1):43. 10.1186/s12889-017-4587-y. 28732487 10.1186/s12889-017-4587-y PMC5521064

[15] D’Souza S, Elshazly MB, Dargham SR, et al. Atrial fibrillation catheter ablation complications in obese and diabetic patients: insights from the US nationwide inpatient sample 2005–2013. Clin Cardiol. 2021;44(8):1151–60. 10.1002/clc.23667. 34132405 10.1002/clc.23667 PMC8364717

[16] Chassin L, Sher KJ. Understanding alcohol use and alcohol use disorders from a developmental psychopathology perspective: research advances, challenges, and future directions. Dev Psychopathol. 2024;36(5):2604–18. 10.1017/S0954579424000671. 38655739 10.1017/S0954579424000671

[17] Gupta NM, Lindenauer PK, Yu PC, et al. Association between alcohol use disorders and outcomes of patients hospitalized with Community-Acquired pneumonia. JAMA Netw Open. 2019;2(6):e195172. 10.1001/jamanetworkopen.2019.5172. Published 2019 Jun 5. 31173120 10.1001/jamanetworkopen.2019.5172 PMC6563577

[18] Pandey S, Bolstad I, Lien L, Bramness JG. Antisocial personality disorder among patients in treatment for alcohol use disorder (AUD): characteristics and predictors of early relapse or drop-out. Subst Abuse Rehabil. 2021;12:11–22. 10.2147/SAR.S296526. 33907489 10.2147/SAR.S296526 PMC8064678

[19] Ferari CS, Katsevman GA, Dekeseredy P, Sedney CL. Elective surgery for acute pain in patients with substance use disorder: lessons learned at a rural neurosurgical center. Patient series. J Neurosurg Case Lessons. 2022;3(13):CASE21656. 10.3171/CASE21656. Published 2022 Mar 28. 36273856 10.3171/CASE21656 PMC9379765

[20] Cheng EP, Liu CF, Yeo I, et al. Risk of mortality following catheter ablation of atrial fibrillation. J Am Coll Cardiol. 2019;74(18):2254–64. 10.1016/j.jacc.2019.08.1036. 31672181 10.1016/j.jacc.2019.08.1036

[21] Chandan N, Ashok V, Hwang T, et al. The influence of risk factor modification on atrial fibrillation outcomes and their impact on the success of catheter ablation. Rev Cardiovasc Med. 2025;26(3):27175. 10.31083/RCM27175. Published 2025 Mar 21. 40160571 10.31083/RCM27175 PMC11951490

[22] Harji DP, Griffiths B, Stocken D, Pearse R, Blazeby J, Brown JM. Protocolized care pathways in emergency general surgery: a systematic review and meta-analysis. Br J Surg. 2024;111(3):znae057. 10.1093/bjs/znae057. 38513265 10.1093/bjs/znae057 PMC10957158

[23] Egholm JW, Pedersen B, Møller AM, Adami J, Juhl CB, Tønnesen H. Perioperative alcohol cessation intervention for postoperative complications. Cochrane Database Syst Rev. 2018;11(11):CD008343. 10.1002/14651858.CD008343.pub3. Published 2018 Nov 8. 30408162 10.1002/14651858.CD008343.pub3 PMC6517044

[24] Barmano N, Charitakis E, Kronstrand R, et al. The association between alcohol consumption, cardiac biomarkers, left atrial size and re-ablation in patients with atrial fibrillation referred for catheter ablation. PLoS One. 2019;14(4):e0215121. 10.1371/journal.pone.0215121. (**Published 2019 Apr 10**). 30970005 10.1371/journal.pone.0215121 PMC6457637

[25] Li R, Huddleston SJ, Prastein DJ. Alcohol use disorder is associated with a lower risk of in-hospital mortality in type A aortic dissection repair: a population-based study of National Inpatient Sample from 2015–2020. Alcohol Alcohol. 2024;59(5):agae061. 10.1093/alcalc/agae061. 39219176 10.1093/alcalc/agae061

[26] Krishnamoorthy P, Kalla A, Figueredo VM. Cardiovascular events in alcoholic syndrome with alcohol withdrawal history: results from the National inpatient sample. Am J Med Sci. 2018;355(5):425–7. 10.1016/j.amjms.2018.01.005. 29753371 10.1016/j.amjms.2018.01.005

[27] Fernandez AC, Chapman L, Ren TY, et al. Preoperative alcohol interventions for elective surgical patients: results from a randomized pilot trial. Surgery. 2022;172(6):1673–81. 10.1016/j.surg.2022.09.012. 36283843 10.1016/j.surg.2022.09.012 PMC10686250

[28] Grindal AW, Sparrow RT, McIntyre WF, Conen D, Healey JS, Wong JA. Alcohol consumption and atrial arrhythmia recurrence after atrial fibrillation ablation: a systematic review and meta-analysis. Can J Cardiol. 2023;39(3):266–73. 10.1016/j.cjca.2022.12.010. 36549481 10.1016/j.cjca.2022.12.010

[29] Butler T, Cowie A, McHale S, Horne S, O’Reilly M, Meelu OA, et al. Interventions for alcohol cessation in people with atrial fibrillation. Cochrane Database Syst Rev. 2023;(2):CD015004. 10.1002/14651858.CD015004.

[30] Asbeutah AA. Alcohol abstinence in drinkers with atrial fibrillation. N Engl J Med. 2020;382(18):1767–8. 10.1056/NEJMc2001512. 32348655 10.1056/NEJMc2001512

[31] Takahashi Y, Nitta J, Kobori A, et al. Alcohol consumption reduction and clinical outcomes of catheter ablation for atrial fibrillation. Circ Arrhythm Electrophysiol. 2021;14(6):e009770. 10.1161/CIRCEP.121.009770. 33999699 10.1161/CIRCEP.121.009770

[32] Cook S, Osborn D, Maini A, et al. Recording of alcohol use disorder in electronic health records: developing a recommended codelist for research. Clin Epidemiol. 2024;16:673–81. 10.2147/CLEP.S477778. 39380579 10.2147/CLEP.S477778 PMC11460355

[33] Laswi H, Abusalim AR, Warraich MS, Khoshbin K, Shaka H. Trends and outcomes of alcoholic liver cirrhosis hospitalizations in the last two decades: analysis of the nationwide inpatient sample. Gastroenterol Res. 2022;15(2):91–9. 10.14740/gr1517. 10.14740/gr1517 PMC9076157 35572473

[34] Han L, Han H, Liu H, et al. Alcohol abuse and alcohol withdrawal are associated with adverse perioperative outcomes following elective spine fusion surgery. Spine. 2021;46(9):588–95. 10.1097/BRS.0000000000003868. 33315773 10.1097/BRS.0000000000003868

[35] Khera R, Angraal S, Couch T, et al. Adherence to methodological standards in research using the National inpatient sample. JAMA. 2017;318(20):2011–8. 10.1001/jama.2017.17653. 29183077 10.1001/jama.2017.17653 PMC5742631

[36] Akano EO, Otite FO, Chaturvedi S. Alcohol withdrawal is associated with poorer outcome in acute ischemic stroke. Neurology. 2019;93(21):e1944–54. 10.1212/WNL.0000000000008518. 31653706 10.1212/WNL.0000000000008518 PMC6885576

