# Supplementary material for: Navigating the challenges of catheter ablation in patients with comorbid alcohol use disorder
Source: BMC Cardiovasc Disord. 2025 Dec 12;26:47. doi: 10.1186/s12872-025-05356-6 (PMC12817500; doi:10.1186/s12872-025-05356-6)
Supplement: Supplementary file 1 — Supplementary Material 1. [file 12872_2025_5356_MOESM1_ESM.docx]

**Table A Study population and catheter ablation code**

|  | ICD-9-CM | ICD-10-CM |
| --- | --- | --- |
| Atrial Fibrillation | 427.31 | I48.0,i48.1,i48.2,i48.91 |
| Catheter ablation procedure | 37.34 | 02583ZZ |
| Atrial flutter | 427.32 | I48.3,i48.4,i48.92 |
| Supraventricular tachycardia | 427.0、427.89、426.7、426.89 | I471 |
| Ventricular tachycardia | 427.1 | I472 |
| Wolff-Parkinson-White syndrome/preexcitation syndrome | 426.7 | I456 |
| other premature beats | 427.69 | I49.4, I49.40, I49.9 |
| cardiac dysrhythmia | 427.89 | I49.9 |
| Alcohol use disorder | 305.00 | F10.10-F10.19 |

**Table B ICD-9 and ICD-10 codes for in-hospital postoperative complications**

| **Diagnosis** | ICD-9-CM | ICD-10-CM |
| --- | --- | --- |
| Myocardial infarction | 412 | I21.A1、I21.A9、I25.2 |
| Atrioventricular block | 426.0 | I44.0、I44.1、I44.2、I44.3 |
| Cardiac arrest | 427.5 | I46.9 |
| Heart failure | 428.1、428.9 | I50.9 |
| Myocardial ischemia | 414.8 | I25.6 |
| Acute myocardial infarction | 410.00 | I21.9 |
| Acute pericarditis | 420.90 | I30.9 |
| Hemopericardium | 423.0 | I31.2 |
| Cardiac tamponade | 423.3 | I31.4 |
| Arteriovenous fistula | 447.0 | I77.0 |
| Vascular damage | 901 | S25 |
| Retroperitoneal injury | 908.1、908.4 | S36.92XS |
| Heart damage | 861.01、861.02 | S26.19XA、S26.19XA |
| Vascular complications of surgery | 997.7 | T81.7 |
| Pneumothorax and hemothorax | 512.1、512.2、860 | J93.9、J94.2 |
| Postoperative respiratory failure | 518.81 | J95.821、J96 |
| Septal muscle paralysis and Phrenic nerve injury | 756.6、519.4 | J98.6 |
| Pulmonary embolism | 41511, 41512, 41513, 41519, | I2601, I2602, I2609, I2690, I2692, I2699 |
| Acute respiratory failure | 51851- 51853 | J95821、J95821 |
| Pneumonia | 480-486 | J12x-J18 |
| Esophagitis | 530.10 | K20.90、K20.91 |
| Esophageal ulcers | 530.21 | K22.10、K22.11 |
| Esophageal strictures | 530.3 | K22.2 |
| Esophageal perforation | 530.4 | K22.3 |
| Gastro-esophageal laceration-hemorrhage syndrome | 530.7 | K22.6 |
| Gastroparesis | 536.3 | K31.84 |
| Gastrointestinal bleeding | 772.4 | K92.2 |
| **Genitourinary disease** | 098.0,098.2 | A54.00 |
| **Acute kidney injury** | 866.00 | N17.9 |
| **Inflammatory diseases of the central nervous system** | 323.02 | G09 |
| **Convulsion** | 345.81,345.91 | R56.9 |
| **Postoperative delirium** | 2930 | F05 |
| **Stroke** | 997.02 | I63.9,G46.4 |
| **Embolism** | 4151x | I82290 |
| **Postoperative shock** | 99800, | T8110XD |
| **Blood transfusion** | 9900-9908 | 30230H1,30230J1,30230K1, 30230L1, 0230M1, |
| **Septicemia** | 0380,0381, 03810-03812 | A400, A401, A403, A408, A409 |
| **Chest pain** | 78650 | R072，R0781, R0782 |
| **Electrolyte imbalance** | 276.9 | E87.8 |
| **Severe malnutrition** | 261,262 | E43,E44.0,E44.1 |
| **Acute respiratory distress syndrome** | 799.1,518.81 | J80,R06.03 |
| **Continuous trauma ventilation** | 9670-9672 | F028GCZ,F028GGZ, F028GYZ, F028GZZ, |
| **Thrombocytopenia** | 287.5 | D69.6 |
| **Liver disease** | 571.1 | K76.9,K70.9 |
| **Hemorrhage/Seroma/Hematoma** | 99811 | I9742 |
